# Genetic determinants of glucose-6-phosphate dehydrogenase activity in Kenya

**DOI:** 10.1186/s12881-014-0093-6

**Published:** 2014-09-09

**Authors:** Shivang S Shah, Alex Macharia, Johnstone Makale, Sophie Uyoga, Katja Kivinen, Rachel Craik, Christina Hubbart, Thomas E Wellems, Kirk A Rockett, Dominic P Kwiatkowski, Thomas N Williams

**Affiliations:** 1 Wellcome Trust Centre for Human Genetics, University of Oxford, Oxford, UK; 2 Kenya Medical Research Institute/Wellcome Trust Research Programme, Centre for Geographic Medicine Research, Coast, Kilifi District Hospital, Kilifi, Kenya; 3 Wellcome Trust Sanger Institute, Hinxton, UK; 4 Nuffield Department of Medicine, John Radcliffe Hospital, University of Oxford, Oxford, UK; 5 Laboratory of Malaria and Vector Research, National Institute of Allergy and Infectious Diseases, National Institutes of Health, Bethesda, Maryland, USA

**Keywords:** G6PD deficiency, Malaria

## Abstract

**Background:**

The relationship between glucose-6-phosphate dehydrogenase (G6PD) deficiency and clinical phenomena such as primaquine-sensitivity and protection from severe malaria remains poorly defined, with past association studies yielding inconsistent and conflicting results. One possibility is that examination of a single genetic variant might underestimate the presence of true effects in the presence of unrecognized functional allelic diversity.

**Methods:**

We systematically examined this possibility in Kenya, conducting a fine-mapping association study of erythrocyte G6PD activity in 1828 Kenyan children across 30 polymorphisms at or around the *G6PD* locus.

**Results:**

We demonstrate a strong functional role for c.202G>A (rs1050828), which accounts for the majority of variance in enzyme activity observed (*P*=1.5×10^−200^, additive model). Additionally, we identify other common variants that exert smaller, intercorrelated effects independent of c.202G>A, and haplotype analyses suggest that each variant tags one of two haplotype motifs that are opposite in sequence identity and effect direction. We posit that these effects are of biological and possible clinical significance, specifically noting that c.376A>G (rs1050829) augments 202AG heterozygote risk for deficiency trait by two-fold (OR = 2.11 [1.12 - 3.84], *P*=0.014).

**Conclusions:**

Our results suggest that c.202G>A is responsible for the majority of the observed prevalence of G6PD deficiency trait in Kenya, but also identify a novel role for c.376A>G as a genetic modifier which marks a common haplotype that augments the risk conferred to 202AG heterozygotes, suggesting that variation at both loci merits consideration in genetic association studies probing G6PD deficiency-associated clinical phenotypes.

## 1 Background

Glucose-6-phosphate dehydrogenase (G6PD) is a housekeeping enzyme encoded on chromosome X. It is ubiquitously expressed in all body tissues and found in eukaryotes and prokaryotes alike. G6PD catalyses the rate-limiting first step in the pentose phosphate pathway, generating NADPH, an electron donor important in a variety of metabolic processes, including fatty and nucleic acid synthesis, nitric oxide production, and the regeneration of antioxidant glutathione. Deficiency of G6PD is the most common enzymopathy in man, with prevalence especially high in sub-Saharan Africa and southeast Asia. It is a trait that is thought to be both harmful and beneficial. While clinical penetrance is generally mild, G6PD deficiency can predispose to hemolytic anemia and neonatal jaundice, generally in the context of oxidant stressors generated by certain foods, medications, and infections. Counterbalancing these deleterious effects is evidence that suggests G6PD deficiency confers partial protection against malaria, and that such protection has conferred a selective advantage to G6PD-deficient individuals in malaria-endemic regions, as reflected in the close geographical correlation between the global distribution of G6PD deficiency and that of historical malaria endemicity.

Despite decades of research, however, our understanding of both beneficial and deleterious clinical phenotypes associated with G6PD deficiency remains incomplete. Association studies of severe malaria have disagreed over the strength and specificity of protection, especially with respect to gender, with past studies demonstrating protection for only males [1], only females [2], both [3],[4], or neither [5]. Additionally, there is geographical heterogeneity in association testing results as well, with the only compelling evidence thus far coming from west Africa. Similarly, the precise determinants of the hemolytic response to oxidative stressors remain unknown. One especially important drug reaction is that involving the antimalarial primaquine [6],[7]. It is currently the only medication capable of radical cure of *Plasmodium vivax* infection and one of few drugs that acts against the *Plasmodium falciparum* gametocyte; it therefore has high value in eliminating reservoirs of infection and reducing transmission rates, tasks that are crucial to the success of any malaria elimination program [8]. While G6PD deficiency alleles are strongly associated with increased risk to primaquine-induced hemolysis, there exists significant unexplained variance in this association, especially for mosaic female heterozygotes [9].

One challenging aspect to designing and interpreting clinical association studies lies in how G6PD deficiency is defined. While many genetic association studies at *G6PD* implicitly assume that one nonsynonymous coding variant has both full positive and negative predictive power for G6PD deficiency trait, this approach belies the considerable allelic heterogeneity present at the locus [4],[10],[11]. Indeed, G6PD deficiency is perhaps best understood as a family of heritable traits exhibiting widely varying prevalence, as well as differential biochemical and clinical penetrance, all factors which influence the power of an association test [4]. Additionally, it is possible that unrecognized genetic modifiers of G6PD activity have yet to be identified, and that these may play a role in determining an individual’s G6PD deficiency status.

To inform our understanding of how genetic variation at *G6PD* impacts clinical outcomes, we sought to examine its more immediate relationship with molecular phenotypes. We took a comprehensive two-part approach, first resequencing the *G6PD* gene to validate known and discover novel variants, and then testing those polymorphisms for association with G6PD enzyme activity in Kenya.

## 2 Methods

### 2.1 Ethics statement

Ethics approval was granted by the Kenya Medical Research Institute (KEMRI) review board, and written informed consent was obtained from a parent or guardian prior to enrollment.

### 2.2 Sample collection

Capillary blood was drawn by heel prick from 2100 healthy infants (less than 6 months old) of predominantly Mijikenda ethnicity, recruited into an ongoing prospective birth cohort study run by KEMRI and the Wellcome Trust in Kilifi, Kenya (SCC1192: The genomic epidemiology of childhood diseases in Kilifi, Kenya). Samples were stored in EDTA at 4°C and processed for activity assays and DNA extraction within four days. Of note, serial assay measurements taken on the same samples remained consistent even up to 10–14 days from collection.

### 2.3 Enzyme activity assay

The assay used is a ten-fold scaled down version of the ‘macro’ assay given in Randox Laboratories G6PD kit (PD 410), using positive (PD 2618) and negative (PD 2617) controls where necessary. Ten microliters of whole blood was washed three times in 100 *μ*l normal saline. Washed RBC were lysed by incubation with 25 *μ*l of digitonin for 15 minutes at 4°C. Five microliters of the hemolysate was incubated with 300 *μ*l of buffer (31.7 mM triethanolamine, 3.2 mM EDTA, pH = 7.6) and 10 *μ*l NADP+ (0.34 mM) at 37°C for 5 minutes. Finally, five microliters of glucose-6-phosphate (0.58 mM) was added and spectrophotometry readings taken, with duplicate measurements taken for each sample. Catalytic rate was assessed by the formation of reaction product NADPH, which absorbs light at 340 nm. The change in absorbance at 340 nm was measured over a 3 minute time course using a plate spectrophotometer (Tecan). As we were unable to obtain hemoglobin or RBC concentrations for our samples to allow for reporting of activity in international units, activity is instead reported here in arbitrary units and was normalized to hemolysate hemoglobin content by recording absorbance at 540 nm:

(1) $$\text{G6PD activity}=\frac{\frac{\mathrm{\Delta OD}340}{\mathrm{\Delta t}}}{\text{OD}540}$$

### 2.4 Sequencing and genotyping

Resequencing was performed as part of an exon sequencing project called ExoSeq at Wellcome Trust Sanger Institute (WTSI). A total of 288 individuals comprised our sample set, with 48 individuals each from Burkina Faso, Cameroon, Nigeria, Kenya, Vietnam, and Papua New Guinea. A total of 28 amplicons approximately 600–800 bp in length were designed via automated algorithm (i.e. in silico PCR), covering about two-thirds of the 15 kb *G6PD* gene, with full coverage of all exons, but a few gaps in noncoding sequence, specifically in introns 1 and 2. Capillary sequencing of the PCR products generated yielded chromatograms that were input into an automated SNP calling pipeline, which was supplemented with manual calls from examination of individual sequence traces. A total of 72 SNPs, including forty-nine novel variants were identified.

Genotyping was performed on the Sequenom®; MASSarray®; iPLEX®; platform. A total of three multiplex genotyping reactions were designed, assaying a total of 68 SNPs. The SNPs input to the design software included those discovered during resequencing (N = 72), as well as nonsynonymous variants from the literature (N = 25), and several hundred variants within 1 Mb of *G6PD* extracted from dbSNP and Ensembl databases. Inclusion in the multiplex reactions was priority-weighted according to predicted functional consequence and estimated minor allele frequency criteria. Template DNA used for genotyping was genomic DNA amplified by primer extension pre-amplification [12]. Genotype calls were manually curated by examining cluster plots to identify ambiguous genotypes. Ancestral and derived alleles were inferred according to the Ensembl EPO pipeline.

### 2.5 Data curation and analysis

Enzyme activity data was filtered to keep only individuals with positive absorbance readings at both 340 and 540 nm, and checked for batch effects and extreme outliers. Genotype data was filtered using custom Perl and R scripts, along with PLINK [13] and snpMatrix [14]. Individuals exhibiting greater than 10% missing data or ambiguous gender (based on chromosome X genotypes) were excluded. SNPs were removed if they showed greater than 5% missing data, minor allele frequency < 1%, or extreme deviation from Hardy-Weinberg equilibrium (*P*<10^−4^, Additional file 1). In total, 1828 of 2100 individuals and 30 out of 70 SNPs met these inclusion criteria (Additional file 2).

Statistical analyses were conducted in R [15]. Single marker tests of association for each derived allele were conducted via multiple linear regression. Covariates included sex, and in the controlled tests, c.202G>A genotype. Three different modes of inheritance are considered– in the additive model, male genotypes were coded 0 or 2, and females were 0, 1, or 2; in the dominant and recessive models, males were coded 0/1, and females were coded 0/1/1, or 0/0/1, respectively. Haplotypes were inferred using fastPHASE [16] under default settings with known male haplotypes used as a reference input to reduce search space and minimize phasing error rate in females (Additional file 3). All haplotypes with frequency > 1% (26 full-length, 13 partial) were tested for association; individuals were coded 0 or 1 based on absence/presence of the test haplotype, with females represented by one haplotype selected at random. Odds ratios (OR) for qualitative tests were calculated via logistic regression. Effect sizes (ES) are presented here with 95% confidence interval in brackets. Allele sharing distance was calculated using a Euclidean distance metric for each individual from both H+ and H- haplotypes. An individual is categorized as either H+ or H- if their distance metric falls into the lowest quintile of the respective empirical distribution. In linear modeling of the additive effect of allele sharing distance on enzyme activity, this categorization is coded as -1 (H-), 0 (neither), or +1 (H+).

## 3 Results

### 3.1 Variants studied

The variants studied here were ascertained using a combination of sources, including our own resequencing efforts, published accounts in the literature, and online variation databases. Resequencing was carried out as part of an ongoing laboratory effort to catalogue global genetic variation at the *G6PD* locus (manuscript in preparation). A total of 15 kb at the *G6PD* locus was sequenced in a worldwide panel comprised of 288 African and Asian individuals, including 48 from Kenya. This work identified a total of 72 SNPs, including 49 novel variants. Published accounts in the literature yielded an additional 25 nonsporadic, nonsynonymous variants associated with G6PD deficiency in Africa and Asia, including 5 that have been identified in sub-Saharan Africa [10]: the c.202G>A, c.376A>G, c.542A>T, c.680G>T, and c.968T>C polymorphisms (number refers to cDNA coordinate). Finally, several hundred polymorphisms (mostly noncoding SNPs) within 1 Mb of *G6PD* were also identified in dbSNP and Ensembl to supplement our list.

This list of several hundred SNPs was then priority-weighted according to predicted functional consequence (e.g. nonsynonymous, splice site, etc.), estimated allele frequency, and proximity to *G6PD* in order to decide which would be prioritized for inclusion in genotyping assays. A total of 68, including all five nonsynonymous African variants, were typed in the current study using the Sequenom®; MASSarray®; iPLEX®; platform. Of the SNPs typed, 30 met final study inclusion criteria, which included having derived allele frequency (DAF) greater than 0.01 and less than 5% missing data. It should be noted that most excluded SNPs failed based on DAF criteria, as many were private to west Africa or Asia. This included the c.542A>T, c.680G>T, and c.968T>C variants, which were monomorphic (DAF = 0%) in Kenya. The only two nonsynonymous variants at the locus that were found to be polymorphic in Kenya were c.202G>A and c.376A>G, exhibiting DAF of 17% and 40%, respectively. All SNPs included in the association study are shown in Additional file 2.

### 3.2 The c.202G>A polymorphism is the major determinant of G6PD activity in Kenya

**Figure 1:**
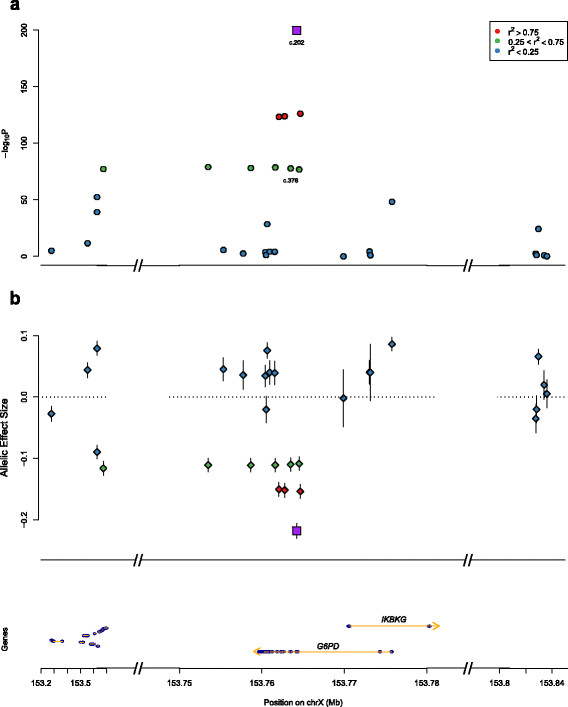
**Fine mapping association study of G6PD activity.a**, P-values and **b**, estimated allelic effect sizes from unbiased association tests (covariate: gender), with different colors indicating strength of linkage disequilibrium with c.202G>A (purple square). Gene graphic shows exons (blue) and introns (yellow) for genes annotated at UCSC Genome Browser (hg19); *IKBKG*=inhibitor of nuclear factor kappa-B kinase subunit gamma.

**Figure 2:**
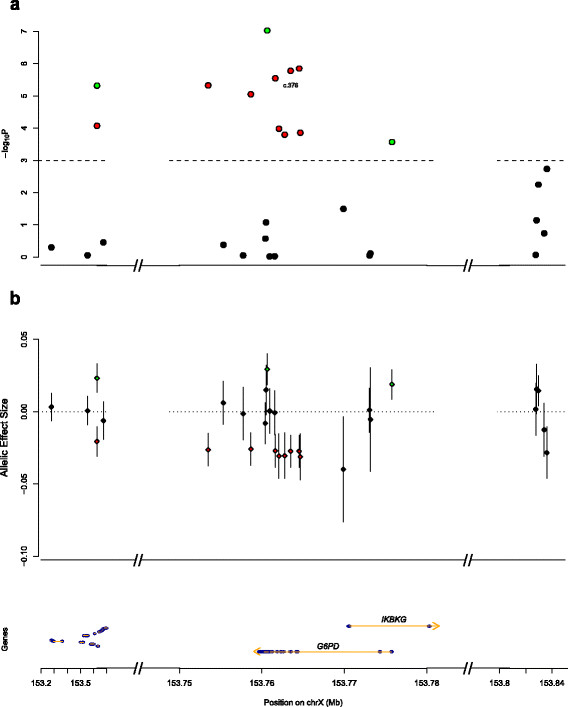
**Association testing for effects independent of c.202G>A.a**, -Log10 P-values and **b**, estimated allelic effect sizes from ‘controlled’ association tests (covariates: gender, c.202G>A genotype). The twelve polymorphisms exhibiting significant effects at *P*<0.001 are colored according to whether they exhibit positive ES (green) or negative ES (red). Gene graphic shows exons (blue) and introns (yellow) for genes annotated at UCSC Genome Browser (hg19); *IKBKG*=inhibitor of nuclear factor kappa-B kinase subunit gamma.

An initial association scan was performed across thirty SNPs in 1828 individuals with only gender included as a covariate. These tests yielded several significant associations (Figure 1, Additional file 4), with the maximum effect size (ES) and lowest P value recorded at c.202G>A (ES = -0.22 [-0.23 - -0.21], *P* = 1.5×10^−200^, additive model), a nonsynonymous coding SNP (rs1050828; Val68Met) known to be associated with G6PD enzyme deficiency in many parts of sub-Saharan Africa. The effect size for the 202A allele corresponds to a roughly 40% reduction in enzyme activity for heterozygotes, and 80% for hemizygotes and homozygotes, consistent with previous estimates [17]-[19]. Since linkage disequilibrium with c.202G>A was highly correlated with signal strength at all other sites, we repeated the association testing including c.202G>A genotype as a covariate. In these controlled tests, we found strong evidence of a causal role for 202A, as all other association signals drop off by many orders of magnitude (Figure 2, Additional file 5), though twelve SNPs still retained significance at *P*<10^−3^, suggesting a role independent of c.202G>A, with allelic ES estimates for each SNP corresponding to 3–4% changes in enzyme activity.

### 3.3 Modifiers of G6PD activity that are independent of c.202G>A

**Figure 3:**
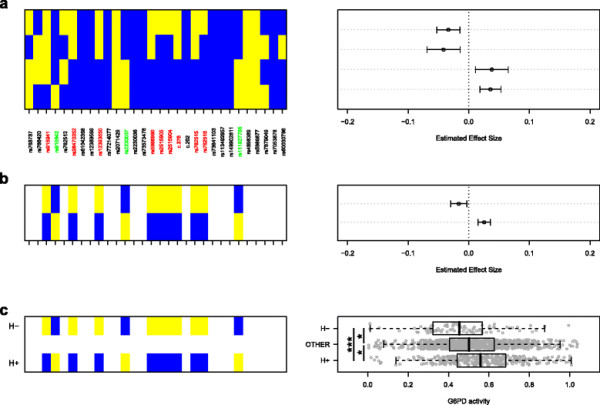
**Haplotype-based association testing for effects independent of c.202G>A.** Results of association testing of **a**, full-length and **b**, partial/twelve-site haplotypes are shown for all haplotype tests suggestive of association (*P*<0.025, covariates: gender, c.202G>A genotype), with haplotypes defined by patterns of ancestral (blue) and derived (yellow) alleles. SNP labels for the twelve sites that comprise the partial haplotypes are colored according to whether they exhibited positive ES [green] or negative ES (red) in single SNP controlled tests (Figure 2). **c**, G6PD activity distribution in c.202G>A wild-type homozygotes (202G or 202GG) as stratified by carriage of H+, H-, or neither haplotype. Pairwise t-test results are shown via asterisk (*: *P*<0.05 and ***: *P*=9.1×10^−5^). In box-and-whisker plot, center of box represents median, edges of box represent upper and lower quartile bounds, and whiskers represent either minimum and maximum values or 1.5 times the interquartile range, whichever is less extreme.

As these twelve SNPs exhibited similar P values and effect size magnitudes (Figure 2, Additional file 5), and were all common variants (DAF > 0.25) at a locus exhibiting a strong pattern of linkage disequilibrium (Additional file 6), we considered the parsimonious hypothesis that they were all related signals. Upon examination of all haplotypes across these twelve sites, it was found that the two most frequent haplotypes (H+ = 19%; H- = 22%) were: (a) exactly opposite in allele identity (Figure 3c), with H+ possessing exactly three derived alleles at sites with positive ES and H- possessing exactly nine derived alleles at the sites with negative ES (Figure 2b), and (b) significantly different in mean enzyme activity in c.202G>A wild-type (i.e. 202GG or 202G) individuals (H+: 0.57 vs. H-: 0.47; *P*=9.1×10^−5^, t-test, Figure 3c). In association testing of all full-length and twelve-site haplotypes, it was observed that the haplotypes most suggestive of association at *P*<0.025 similarly exhibited differential ES direction and resembled either H+ or H- in sequence identity (Figure 3a and b, Additional file 7). Additionally, the extent of allele sharing with either the H+ or H- haplotype was correlated with enzyme activity independent of c.202G>A (*P*=2.3×10^−6^, additive linear model, Additional file 8). Lastly, it was noted that SNPs tagging the H+ (rs2230037) and H- (c.376A>G) haplotypes, in concert with c.202G>A, were sufficient to describe a minimal adequate genetic model of G6PD activity that could explain 41% of variance in enzyme activity (Table 1).

**Table 1:** Minimal adequate genetic model of G6PD activity

**SNP**	**Allelic ES (95% CI)**	**% change**
c.202	-0.198 (-0.213 - -0.183)	-37.9
rs2230037 (H+)	+0.022 (+0.011 - +0.034)	4.3
c.376 (H-)	-0.017 (-0.030 - -0.005)	-3.3

A linear model containing both c.202G>A as well as SNPs tagging the H+ (rs2230037) and H- (c.376A>G) haplotypes explained significantly more variance than a model containing c.202G>A alone (*P****<***0.001), and could not be improved via the inclusion of additional SNPs.

While the association of c.376A>G with a modest reduction in enzyme activity (approximately 10–20% in male hemizygotes) has been reported previously [20],[21], and this has been generally thought to be the result of changes to protein structure leading to enzyme instability, it should be emphasized that neither the controlled association scan nor subsequent haplotype testing was suggestive of a uniquely causal role for c.376A>G. Indeed, what our work suggests instead is the possibility that this nonsynonymous variant is merely a marker, as it is only one of nine SNPs tagging the H- haplotype and one of twelve overall that exhibits modest effects independent of c.202G>A.

### 3.4 Both c.202G>A and c.376A>G contribute to risk of G6PD deficiency state

**Figure 4:**
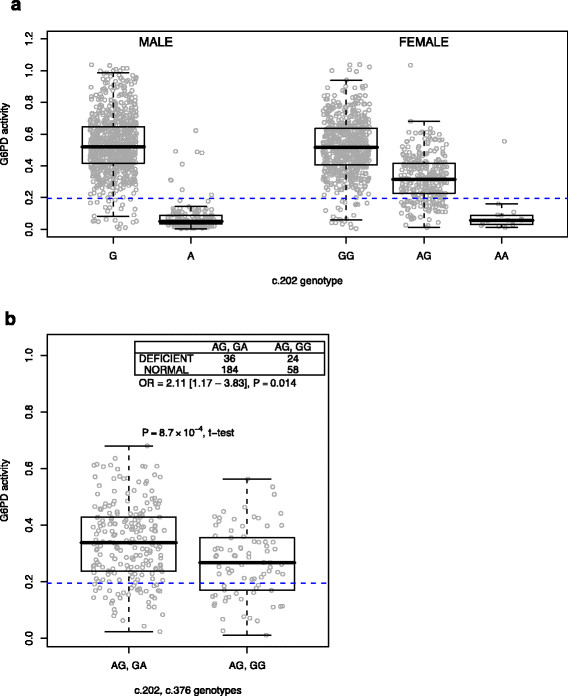
**Genetic determinants of G6PD deficiency status.a**, G6PD activity distribution stratified by genotype at c.202G>A. **b**, G6PD activity in 202AG heterozygotes stratified by genotype at c.376A>G (NB: the 202A derived allele always exists on a 376G derived allele background), with contingency table (inset) for qualitative test of association at c.376A>G in 202AG heterozygotes. Also shown in both plots is the reference line (blue) dividing normal vs. deficient for qualitative tests of G6PD deficiency. In box-and-whisker plots, center of box represents median, edges of box represent upper and lower quartile bounds, and whiskers represent either minimum and maximum values or 1.5 times the interquartile range, whichever is less extreme.

We also examined qualitative G6PD activity status, using the intermodal trough of the bimodal activity distribution in males as an ad hoc cutoff to distinguish normal from deficient (Figure 4A, Additional file 9). Overall, using the clearer distribution profile in males, we estimate that the prevalence of G6PD deficiency in our cohort is 18%, and that 202A accounts for 85% of this figure, which suggests that there may be rare functional variants we were underpowered to discover, consistent with observations elsewhere in east Africa [19]. A total of 162 males and 97 females could be categorized as deficient, including 93% of both 202A hemizygote males and 202AA homozygote females, but notably also 20% of 202AG heterozygotes.

While it is known that the distribution of enzyme activity in 202AG female heterozygotes is broad, with some individuals exhibiting an effectively deficient biochemical phenotype, little is known about the genetic factors that might contribute to this risk. We tested the possibility that our H- tag SNP, c.376A>G, might reduce enzyme activity sufficiently to augment risk of phenotypic G6PD deficiency state in such individuals. The 202A allele is known to obligately lie on a 376G background in African populations to form the ‘A-’ haplotype [17],[22],[23], and thus 202AG individuals can be either 376GA or 376GG. We found that among 202AG heterozygotes, those that were 376GG exhibited not only reduced enzyme activity as compared to 376GA (ES = -0.06 [-0.10 - -0.03], *P*=0.0009, Figure 4b), a trend consistent with that observed earlier in 202 wild-type individuals, but were also notably at increased risk for deficiency state (OR = 2.11 [1.12 - 3.84], *P*=0.014). This latter finding has not been previously reported, and represents the first demonstration of a genetic modifier that influences risk to deficiency state in individuals heterozygous for the A- form of G6PD deficiency.

## 4 Discussion

The work presented here represents the first fine-mapping association study of enzyme activity at the *G6PD* locus, and provides new insights into the genetic basis for quantitative and qualitative variation in this clinically-important molecular phenotype. We found that the largest proportion of variance in G6PD activity could be explained by a single polymorphism, c.202G>A, a coding variant thought to be a target of recent positive selection [24],[25], but one that has yielded conflicting results in previous association studies of severe malaria. In addition, we found that certain common variants at the locus modulate G6PD activity independent of c.202G>A, and identify among these a role for c.376A>G as a genetic modifier which augments by two-fold the risk of G6PD deficiency state in 202AG heterozygote females. Taken together, these results suggest that future clinical association studies in Africa should consider the effects of both of these variants in order to arrive at a more accurate genetic definition of phenotypic G6PD deficiency state.

Our study is the first to systematically consider the possibility that other SNPs, even those found several hundred kilobases away from *G6PD*, might be associated with G6PD deficiency, and that c.202G>A might only be indirectly associated with deficiency, a finding which could explain discrepancies in previous association studies. Although the number of SNPs surveyed here is relatively small and does not include rare variants, we did not observe any SNPs more strongly associated with G6PD deficiency in Kenya than the c.202G>A variant, strongly suggestive of a causal role. Additionally, while all known functional variants in sub-Saharan Africa were genotyped as part of our study, only c.202G>A was found to be polymorphic in Kenya (DAF = 17%). This allelic homogeneity contrasts with the situation in some areas of west Africa [4], and suggests that given a similar allele frequency and biochemical penetrance, clinical association studies of malaria in each region should be equivalently powered if indeed the G6PD deficiency trait is directly responsible for protection. The strongest evidence implicating G6PD deficiency in malaria protection would ideally be consistent with an additive genetic model, with hemizygous males and homozygous females exhibiting similar clinical phenotypes, and heterozygous females displaying intermediate phenotypes.

Our work also demonstrates the power of a haplotype-based approach in resolving multiple correlated association signals in a region. Though we found two haplotype motifs that were common and of significant effect independent of c.202G>A, we were unable to further localize the signal, even when examining all possible two- and three-locus haplotypes (data not shown), suggesting that these haplotypes are merely markers for the true causal variants. Interestingly, among the twelve variants of significant effect independent of c.202G>A were c.376A>G, a nonsynonymous coding variant, as well as rs915942 and rs111827785, predicted 5’ UTR splice-site variants within *RPL10* (a large-subunit ribosomal gene) and *G6PD*, respectively. While we can only speculate as to the precise nature of the structure-function relationship mediating these effects, the biology of the locus makes for interesting possibilities for interaction between c.202G>A and the H+/H- variants, including the possibility of cell-cell interactions synergistically impacting enzyme activity distribution in 202AG mosaic females.

At one level, the work presented here reveals how common, standing genetic variation superimposed on a Mendelian trait can significantly impact expression of that trait. More important, however, is how this finding might translate into a better understanding of how G6PD deficiency is linked to key clinical phenomena. Specifically, it will be worth considering whether two site genetic testing is more predictive of clinical outcome than single SNP testing in women heterozygous for the common A- form of G6PD deficiency in Africa. For example, in considering a public health intervention such as mass primaquine administration as part of a malaria elimination campaign, two-site testing might help to identify the subset of A- heterozygotes more likely to be susceptible to acute hemolytic anemia. Lastly, at a more general level, our work highlights the importance of measuring intermediate molecular phenotypes that can bridge the gulf between variance at the genetic level and variance in clinical phenotypes. This is especially important for association studies of G6PD deficiency given its considerable allelic heterogeneity, varying biochemical penetrance, and long history of inconsistent findings.

## 5 Conclusions

We have surveyed genetic variation at the G6PD locus in Kenya via resequencing, and conducted a fine-mapping association study of erythrocyte G6PD activity. We find that while the c.202G>A polymorphism accounts for most of the variance in enzyme activity seen in this population, several other common polymorphisms are also relevant, specifically noting that c.376A>G acts as a genetic modifier which increases by two-fold the risk of deficiency state in 202AG heterozygotes. Our results suggest that multiple alleles at the G6PD locus play a role in determining G6PD deficiency state and therefore merit consideration in genetic association studies of clinical phenotypes such as severe malaria or drug sensitivity.

## Competing interests

The authors declare that they have no competing interests.

## Authors’ contributions

SS, KR, TEW, DPK and TNW designed the study. SS and KR designed and validated the enzyme activity and genotyping assays. AM, JM and SU performed enzyme activity assays. SS, RC and CH performed genotyping. KK performed sequencing. SS conducted statistical analyses. SS, KR, TEW, DPK and TNW wrote the paper. All authors read and approved the final manuscript.

## Additional files

## Supplementary Material

Additional file 1 **Quality control plots.** Shown here are plots that examine: (a) whether any individuals with low call rate and high heterozygosity (left), or (b) whether any SNPs with low call rate and extreme deviation from HWE (right) could be found, features indicative of low confidence samples and SNPs, respectively. Click here for file

Additional file 2 **SNP summary table.** Shown here for each SNP assayed is: the genomic position (hg19/GRCh37), observed alleles (on the [+]-strand, according to UCSC Genome Browser), ancestral allele (according to the Ensembl EPO pipeline), G6PD exon-intron position (according to UCSC genome annotation coordinates), and derived allele frequency stratified by sex. Key: E=exon, I=intron, US=upstream, DS=downstream. NB: c.202 and c.376 are referred to by their coding strand (-) allele designations throughout for clarity. Click here for file

Additional file 3 **Haplotype distribution in males vs. females.** The frequency of each haplotype is plotted in decreasing order, stratified by sex (top, males=dark bars, females=light bars). No significant differences between male and female haplotype frequencies were found, attesting to highly accurate haplotype phasing (bottom). Click here for file

Additional file 4 **Initial association test results.** Shown here for each SNP surveyed is: genomic position (hg19/GRCh37), DAF stratified by sex, P values and effect sizes (with 95% CI) for initial, uncontrolled association tests under three different genetic models. Click here for file

Additional file 5 **Association test results for effects independent of c.202G>A.** Shown here for each SNP surveyed is: genomic position (hg19/GRCh37), DAF stratified by sex, P values and effect sizes (with 95% CI) for c.202G>A-controlled association tests under three different genetic models. Click here for file

Additional file 6 **Linkage disequilibrium (LD) across region.** Pairwise LD matrix is shown in colorimetric form, using the D’ and LOD metrics. Colors indicate: D’ = 1 and LOD ≥ 2 (red), D’ = 1 and LOD ≤ 2 (blue), D’ < 1 and LOD ≥ 2 (pink), D’ < 1 and LOD ≤ 2 (white). Click here for file

Additional file 7 **Association test results of full-length and partial haplotypes for effects independent of c.202G>A.** Shown here for each haplotype surveyed is: allele identity across all sites (0=ancestral, 1=derived, _=either), population frequency, P values and effect sizes (with 95% CI) for c.202G>A-controlled association tests. Click here for file

Additional file 8 **Allele sharing with H+ or H- explains variance independent of c.202G>A.** G6PD activity as stratified by sex and haplogroup, where the latter is defined by categorizing each individual according to extensive allele sharing with either the H+, H-, or neither haplotype (see Methods). In box-and-whisker plots, center of box represents median, edges of box represent upper and lower quartile bounds, and whiskers represent either minimum and maximum values or 1.5 times the interquartile range, whichever is less extreme. The P-value displayed in the figure represents the statistical significance of allele sharing under a linear model which incorporates c.202G>A genotype and gender as covariates (NB: sex-stratified testing reveals *P*_*male*_=8.8×10^−5^ and *P*_*female*_=6.2×10^−4^). Click here for file

Additional file 9 **Defining qualitative G6PD deficiency.** The intermodal minimum in the G6PD activity distribution in males was determined using kernel density estimation of the probability density function (red) and provided our ad hoc cutoff (blue) for distinguishing normal vs. deficient. A histogram of the G6PD activity distribution in males is superimposed for clarity. Click here for file
