# Supplementary material for: Genetic determinants of glucose-6-phosphate dehydrogenase activity in Kenya
Source: BMC Med Genet. 2014 Sep 9;15:93. doi: 10.1186/s12881-014-0093-6 (PMC4236593; doi:10.1186/s12881-014-0093-6)
Supplement: Additional file 1 — Quality control plots. Shown here are plots that examine: (a) whether any individuals with low call rate and high heterozygosity (left), or (b) whether any SNPs with low call rate and extreme deviation from HWE (right) could be found, features indicative of low confidence samples and SNPs, respectively. [file s12881-014-0093-6-S1.pdf]

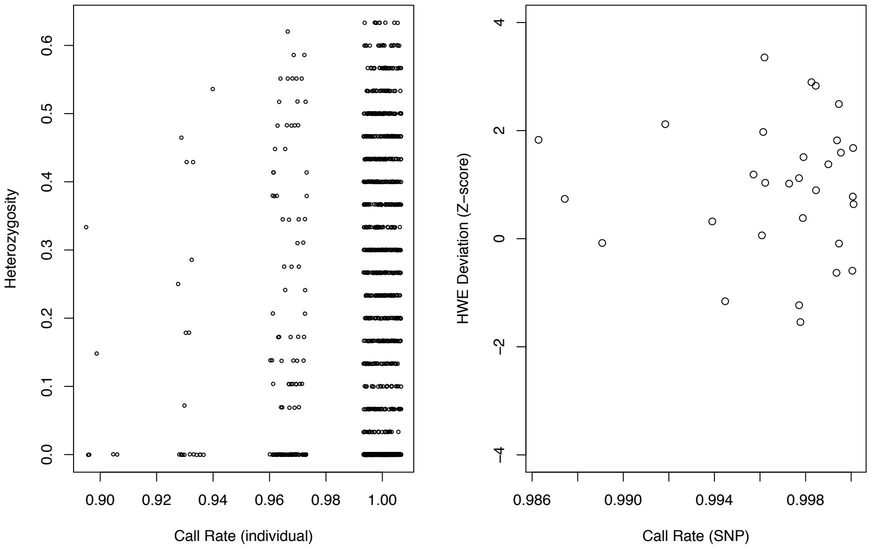

Figure S1. Quality control plots. Shown here are plots that examine: (a) whether any individuals with low call rate and high heterozygosity (left), or (b) whether any SNPs with low call rate and extreme deviation from HWE (right) could be found, features indicative of low confidence samples and SNPs, respectively.
