# Supplementary material for: Genetic determinants of glucose-6-phosphate dehydrogenase activity in Kenya
Source: BMC Med Genet. 2014 Sep 9;15:93. doi: 10.1186/s12881-014-0093-6 (PMC4236593; doi:10.1186/s12881-014-0093-6)
Supplement: Additional file 2 — SNP summary table. Shown here for each SNP assayed is: the genomic position (hg19/GRCh37), observed alleles (on the [+]-strand, according to UCSC Genome Browser), ancestral allele (according to the Ensembl EPO pipeline), G6PD exon-intron position (according to UCSC genome annotation coordinates), and derived allele frequency stratified by sex. Key: E=exon, I=intron, US=upstream, DS=downstream. NB: c.202 and c.376 are referred to by their coding strand (-) allele designations throughout for clarity. [file s12881-014-0093-6-S2.pdf]

Table S1. SNP summary table. Shown here for each SNP assayed is: the genomic position (hg19/GRCh37), observed alleles (on the [+]-strand, according to UCSC Genome Browser), ancestral allele (according to the Ensembl EPO pipeline), G6PD exon-intron position (according to UCSC genome annotation coordinates), and derived allele frequency stratified by sex. Key: E=exon, I=intron, US=upstream, DS=downstream. NB: c.202 and c.376 are referred to by their coding strand (-) allele designations throughout for clarity.

Name	Position (build 37)	Observed Alleles	Ancestral Allele	G6PD - Exon/Intron	DAF (male)	DAF (female)
rs763737	153278307	G/A	G	DS	0.611	0.607
rs766420	153554404	G/C	G	DS	0.361	0.321
rs915941	153626649	C/A	C	DS	0.481	0.462
rs915942	153626738	G/A	G	DS	0.421	0.429
rs762513	153675171	A/G	A	DS	0.310	0.311
rs28470352	153753490	T/A	T	DS	0.391	0.391
rs61042368	153755336	G/A	G	DS	0.111	0.110
rs12389569	153757734	G/A	G	DS	0.067	0.072
rs12393550	153758660	G/A	G	DS	0.390	0.387
rs77214077	153760429	G/A	G	E12	0.129	0.113
rs2071429	153760508	A/G	A	I11	0.925	0.921
rs2230037	153760654	G/A	G	E11	0.253	0.265
rs2230036	153760953	C/T	C	E10	0.104	0.098
rs73573478	153761564	G/A	G	I8	0.105	0.103
rs5986990	153761628	G/A	G	I8	0.391	0.392
rs2515905	153762075	G/A	G	I7	0.269	0.259
rs2515904	153762771	G/C	G	I5	0.265	0.256
c.376 (rs1050829)	153763492	T/C	T	E5	0.400	0.395
c.202 (rs1050828)	153764217	C/T	C	E4	0.162	0.183
rs762515	153764528	T/C	T	I2	0.403	0.400
rs762516	153764663	C/T	C	I2	0.262	0.253
rs73641103	153769889	G/A	G	I2	0.018	0.012
rs113492957	153773062	C/T	C	I2	0.105	0.101
rs149902811	153773160	A/C	A	I2	0.014	0.021
rs111827785	153775785	T/C	C	US	0.488	0.484
rs4898389	153827637	A/G	A	US	0.935	0.928
rs5986877	153828269	C/G	C	US	0.926	0.918
rs7879049	153829693	A/G	A	US	0.323	0.323
rs7053878	153834100	T/A	T	US	0.063	0.072
rs60030796	153836171	A/G	A	US	0.073	0.068
