# Supplementary material for: Genetic determinants of glucose-6-phosphate dehydrogenase activity in Kenya
Source: BMC Med Genet. 2014 Sep 9;15:93. doi: 10.1186/s12881-014-0093-6 (PMC4236593; doi:10.1186/s12881-014-0093-6)
Supplement: Additional file 3 — Haplotype distribution in males vs. females. The frequency of each haplotype is plotted in decreasing order, stratified by sex (top, males=dark bars, females=light bars). No significant differences between male and female haplotype frequencies were found, attesting to highly accurate haplotype phasing (bottom). [file s12881-014-0093-6-S3.pdf]

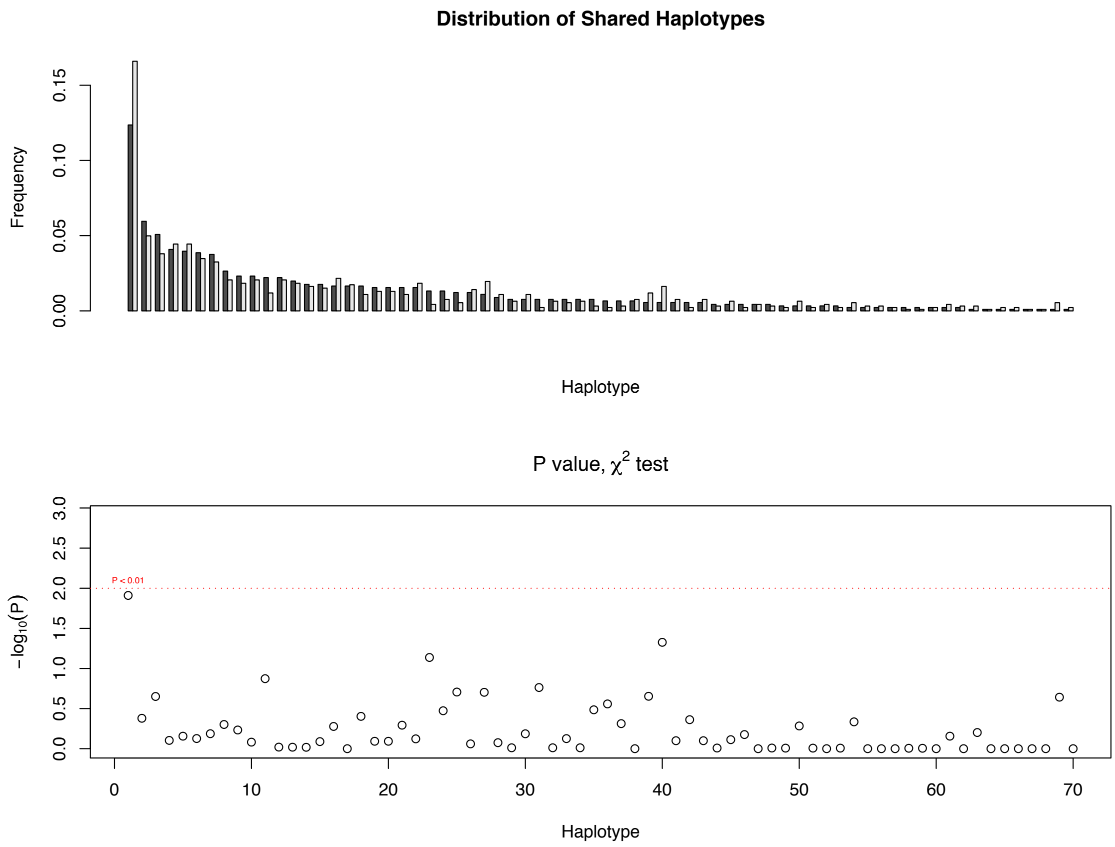

Figure S2. Haplotype distribution in males vs. females. The frequency of each haplotype is plotted in decreasing order, stratified by sex (top). No significant differences between male and female haplotype frequencies were found, attesting to highly accurate haplotype phasing (bottom).
