# Supplementary material for: Genetic determinants of glucose-6-phosphate dehydrogenase activity in Kenya
Source: BMC Med Genet. 2014 Sep 9;15:93. doi: 10.1186/s12881-014-0093-6 (PMC4236593; doi:10.1186/s12881-014-0093-6)
Supplement: Additional file 4 — Initial association test results. Shown here for each SNP surveyed is: genomic position (hg19/GRCh37), DAF stratified by sex, P values and effect sizes (with 95% CI) for initial, uncontrolled association tests under three different genetic models. [file s12881-014-0093-6-S4.pdf]

		DAF		Additive		Dominant		Recessive	
	Position	Male	Female	P	Effect Size	P	Effect Size	P	Effect Size
c.202	153764217	0.162	0.183	1.5e-200	-0.22 (-0.23 - -0.21)	8.1e-163	-0.30 (-0.32 - -0.28)	2.8e-147	-0.44 (-0.47 - -0.41)
rs762516	153764663	0.262	0.253	5.5e-127	-0.15 (-0.17 - -0.14)	9.7e-110	-0.23 (-0.25 - -0.21)	1.1e-96	-0.30 (-0.32 - -0.27)
rs2515904	153762771	0.265	0.256	1.2e-124	-0.15 (-0.16 - -0.14)	1.5e-107	-0.23 (-0.24 - -0.21)	2.0e-95	-0.29 (-0.32 - -0.27)
rs2515905	153762075	0.269	0.259	3.7e-124	-0.15 (-0.16 - -0.14)	1.4e-108	-0.23 (-0.24 - -0.21)	1.2e-93	-0.29 (-0.31 - -0.26)
rs28470352	153753490	0.391	0.391	1.0e-79	-0.11 (-0.12 - -0.10)	6.4e-69	-0.18 (-0.20 - -0.16)	2.9e-65	-0.20 (-0.22 - -0.18)
rs5986990	153761628	0.391	0.392	1.9e-79	-0.11 (-0.12 - -0.10)	1.2e-68	-0.18 (-0.20 - -0.16)	6.1e-65	-0.20 (-0.22 - -0.18)
rs12393550	153758660	0.390	0.387	6.4e-79	-0.11 (-0.12 - -0.10)	1.1e-69	-0.18 (-0.20 - -0.16)	1.2e-62	-0.20 (-0.22 - -0.17)
c.376	153763492	0.400	0.395	1.4e-78	-0.11 (-0.12 - -0.10)	4.0e-68	-0.18 (-0.20 - -0.16)	3.0e-64	-0.20 (-0.22 - -0.17)
rs762513	153675171	0.310	0.311	5.0e-78	-0.12 (-0.13 - -0.10)	9.8e-73	-0.18 (-0.20 - -0.17)	2.3e-56	-0.21 (-0.23 - -0.18)
rs762515	153764528	0.403	0.400	1.2e-77	-0.11 (-0.12 - -0.10)	1.3e-68	-0.18 (-0.20 - -0.16)	3.6e-62	-0.19 (-0.21 - -0.17)
rs915941	153626649	0.481	0.462	3.7e-53	-0.09 (-0.10 - -0.08)	3.4e-47	-0.15 (-0.17 - -0.13)	3.0e-43	-0.15 (-0.17 - -0.13)
rs111827785	153775785	0.488	0.484	4.2e-49	+0.09 (+0.07 - +0.10)	4.5e-41	+0.14 (+0.12 - +0.16)	1.2e-42	+0.15 (+0.13 - +0.17)
rs915942	153626738	0.421	0.429	6.4e-40	+0.08 (+0.07 - +0.09)	2.2e-33	+0.13 (+0.11 - +0.15)	8.1e-35	+0.14 (+0.12 - +0.17)
rs2230037	153760654	0.253	0.265	2.4e-29	+0.08 (+0.06 - +0.09)	3.4e-25	+0.11 (+0.09 - +0.13)	1.9e-24	+0.15 (+0.12 - +0.17)
rs7879049	153829693	0.323	0.323	5.0e-25	+0.07 (+0.05 - +0.08)	1.3e-20	+0.10 (+0.08 - +0.12)	3.1e-22	+0.13 (+0.10 - +0.15)
rs766420	153554404	0.361	0.321	2.2e-12	+0.04 (+0.03 - +0.06)	4.0e-11	+0.07 (+0.05 - +0.09)	2.5e-10	+0.08 (+0.06 - +0.11)
rs61042368	153755336	0.111	0.110	2.1e-06	+0.05 (+0.03 - +0.06)	8.6e-06	+0.06 (+0.04 - +0.09)	5.1e-05	+0.09 (+0.05 - +0.13)
rs763737	153278307	0.611	0.607	9.0e-06	-0.03 (-0.04 - -0.02)	5.5e-06	-0.05 (-0.08 - -0.03)	2.2e-04	-0.04 (-0.06 - -0.02)
rs113492957	153773062	0.105	0.101	4.3e-05	+0.04 (+0.02 - +0.06)	1.2e-04	+0.06 (+0.03 - +0.08)	5.3e-04	+0.08 (+0.03 - +0.13)
rs2230036	153760953	0.104	0.098	5.5e-05	+0.04 (+0.02 - +0.06)	1.8e-04	+0.05 (+0.03 - +0.08)	4.7e-04	+0.08 (+0.04 - +0.13)
rs73573478	153761564	0.105	0.103	7.3e-05	+0.04 (+0.02 - +0.06)	2.5e-04	+0.05 (+0.02 - +0.08)	5.3e-04	+0.08 (+0.03 - +0.13)
rs77214077	153760429	0.129	0.113	1.5e-04	+0.03 (+0.02 - +0.05)	1.4e-04	+0.05 (+0.03 - +0.08)	3.3e-03	+0.06 (+0.02 - +0.10)
rs12389569	153757734	0.067	0.072	2.6e-03	+0.04 (+0.01 - +0.06)	1.3e-02	+0.04 (+0.01 - +0.08)	1.9e-03	+0.09 (+0.03 - +0.14)
rs4898389	153827637	0.935	0.928	3.0e-03	-0.04 (-0.06 - -0.01)	7.1e-02	-0.05 (-0.11 - +0.00)	1.3e-03	-0.05 (-0.09 - -0.02)
rs2071429	153760508	0.925	0.921	6.8e-02	-0.02 (-0.04 - +0.00)	2.5e-01	-0.03 (-0.08 - +0.02)	5.0e-02	-0.03 (-0.06 - -0.00)
rs5986877	153828269	0.926	0.918	7.1e-02	-0.02 (-0.04 - +0.00)	2.6e-01	-0.03 (-0.08 - +0.02)	5.1e-02	-0.03 (-0.06 - +0.00)
rs149902811	153773160	0.014	0.021	8.9e-02	+0.04 (-0.01 - +0.09)	7.9e-02	+0.06 (-0.01 - +0.12)	3.2e-01	+0.06 (-0.06 - +0.18)
rs7053878	153834100	0.063	0.072	1.0e-01	+0.02 (-0.00 - +0.04)	1.7e-01	+0.02 (-0.01 - +0.06)	9.9e-02	+0.05 (-0.01 - +0.10)
rs60030796	153836171	0.073	0.068	6.5e-01	+0.01 (-0.02 - +0.03)	8.6e-01	-0.00 (-0.04 - +0.03)	1.8e-01	+0.04 (-0.02 - +0.09)
rs73641103	153769889	0.018	0.012	9.3e-01	-0.00 (-0.05 - +0.04)	8.7e-01	-0.01 (-0.08 - +0.06)	9.6e-01	+0.00 (-0.11 - +0.11)

Table S2. Initial association test results. Shown here for each SNP surveyed is: genomic position (hg19/GRCh37), DAF stratified by sex, P values and effect sizes (with 95% CI) for initial, uncontrolled association tests under three different genetic models.
