# Supplementary material for: Genetic determinants of glucose-6-phosphate dehydrogenase activity in Kenya
Source: BMC Med Genet. 2014 Sep 9;15:93. doi: 10.1186/s12881-014-0093-6 (PMC4236593; doi:10.1186/s12881-014-0093-6)
Supplement: Additional file 5 — Association test results for effects independent of c.202G>A. Shown here for each SNP surveyed is: genomic position (hg19/GRCh37), DAF stratified by sex, P values and effect sizes (with 95% CI) for c.202G>A-controlled association tests under three different genetic models. [file s12881-014-0093-6-S5.pdf]

		DAF		Additive		Dominant		Recessive	
	Position	Male	Female	P	Effect Size	P	Effect Size	P	Effect Size
rs2230037	153760654	0.253	0.265	9.3e-08	+0.03 (+0.02 - +0.04)	1.3e-07	+0.05 (+0.03 - +0.07)	6.2e-10	+0.08 (+0.05 - +0.10)
rs762515	153764528	0.403	0.400	1.4e-06	-0.03 (-0.04 - -0.02)	1.5e-07	-0.05 (-0.07 - -0.03)	2.0e-11	-0.08 (-0.10 - -0.05)
c.376	153763492	0.400	0.395	1.7e-06	-0.03 (-0.04 - -0.02)	4.4e-07	-0.05 (-0.07 - -0.03)	4.6e-12	-0.08 (-0.10 - -0.06)
rs5986990	153761628	0.391	0.392	2.8e-06	-0.03 (-0.04 - -0.02)	7.0e-07	-0.05 (-0.07 - -0.03)	5.9e-12	-0.08 (-0.10 - -0.06)
rs28470352	153753490	0.391	0.391	4.7e-06	-0.03 (-0.04 - -0.02)	1.0e-06	-0.05 (-0.07 - -0.03)	1.2e-11	-0.08 (-0.10 - -0.06)
rs915942	153626738	0.421	0.429	4.8e-06	+0.02 (+0.01 - +0.03)	2.0e-08	+0.05 (+0.03 - +0.07)	6.4e-12	+0.07 (+0.05 - +0.09)
rs12393550	153758660	0.390	0.387	8.9e-06	-0.03 (-0.04 - -0.01)	4.5e-07	-0.05 (-0.07 - -0.03)	2.0e-10	-0.07 (-0.10 - -0.05)
rs915941	153626649	0.481	0.462	8.4e-05	-0.02 (-0.03 - -0.01)	4.7e-06	-0.04 (-0.06 - -0.03)	8.3e-09	-0.06 (-0.08 - -0.04)
rs2515905	153762075	0.269	0.259	1.0e-04	-0.03 (-0.05 - -0.02)	1.6e-04	-0.05 (-0.08 - -0.02)	2.1e-06	-0.08 (-0.11 - -0.05)
rs762516	153764663	0.262	0.253	1.4e-04	-0.03 (-0.05 - -0.02)	3.2e-04	-0.05 (-0.08 - -0.02)	5.4e-06	-0.08 (-0.12 - -0.05)
rs2515904	153762771	0.265	0.256	1.6e-04	-0.03 (-0.05 - -0.01)	3.5e-04	-0.05 (-0.08 - -0.02)	3.2e-06	-0.08 (-0.12 - -0.05)
rs111827785	153775785	0.488	0.484	2.7e-04	+0.02 (+0.01 - +0.03)	9.1e-08	+0.05 (+0.03 - +0.07)	6.7e-13	+0.07 (+0.05 - +0.09)
rs60030796	153836171	0.073	0.068	1.8e-03	-0.03 (-0.05 - -0.01)	6.8e-03	-0.04 (-0.07 - -0.01)	1.0e-01	-0.04 (-0.08 - +0.01)
rs7879049	153829693	0.323	0.323	5.6e-03	+0.01 (+0.00 - +0.02)	7.2e-04	+0.03 (+0.01 - +0.05)	7.1e-06	+0.05 (+0.03 - +0.07)
rs73641103	153769889	0.018	0.012	3.2e-02	-0.04 (-0.08 - -0.00)	4.9e-02	-0.06 (-0.11 - -0.00)	1.3e-01	-0.07 (-0.16 - +0.02)
rs5986877	153828269	0.926	0.918	7.2e-02	+0.02 (-0.00 - +0.03)	1.7e-01	+0.03 (-0.01 - +0.07)	9.8e-01	+0.00 (-0.03 - +0.03)
rs2071429	153760508	0.925	0.921	8.4e-02	+0.02 (-0.00 - +0.03)	1.8e-01	+0.03 (-0.01 - +0.07)	9.7e-01	+0.00 (-0.03 - +0.03)
rs7053878	153834100	0.063	0.072	1.8e-01	-0.01 (-0.03 - +0.01)	5.2e-01	-0.01 (-0.04 - +0.02)	3.4e-01	-0.02 (-0.07 - +0.02)
rs77214077	153760429	0.129	0.113	2.7e-01	-0.01 (-0.02 - +0.01)	6.1e-01	-0.01 (-0.03 - +0.02)	3.9e-01	-0.02 (-0.05 - +0.02)
rs762513	153675171	0.310	0.311	3.5e-01	-0.01 (-0.02 - +0.01)	5.5e-02	-0.02 (-0.04 - +0.00)	6.9e-02	-0.03 (-0.05 - +0.00)
rs61042368	153755336	0.111	0.110	4.1e-01	+0.01 (-0.01 - +0.02)	2.2e-01	+0.01 (-0.01 - +0.04)	4.8e-01	+0.01 (-0.02 - +0.05)
rs763737	153278307	0.611	0.607	5.0e-01	+0.00 (-0.01 - +0.01)	6.8e-01	-0.00 (-0.02 - +0.02)	7.2e-01	-0.00 (-0.02 - +0.01)
rs149902811	153773160	0.014	0.021	7.7e-01	-0.01 (-0.04 - +0.03)	6.0e-01	-0.01 (-0.06 - +0.04)	8.2e-01	-0.01 (-0.11 - +0.09)
rs4898389	153827637	0.935	0.928	8.5e-01	+0.00 (-0.02 - +0.02)	7.9e-01	+0.01 (-0.04 - +0.05)	9.9e-02	-0.02 (-0.05 - +0.00)
rs766420	153554404	0.361	0.321	8.8e-01	+0.00 (-0.01 - +0.01)	3.9e-01	+0.01 (-0.01 - +0.02)	7.4e-02	+0.02 (-0.00 - +0.04)
rs12389569	153757734	0.067	0.072	8.8e-01	-0.00 (-0.02 - +0.02)	7.6e-01	-0.00 (-0.03 - +0.02)	4.4e-01	+0.02 (-0.03 - +0.06)
rs113492957	153773062	0.105	0.101	8.9e-01	+0.00 (-0.01 - +0.02)	5.7e-01	+0.01 (-0.02 - +0.03)	9.2e-01	+0.00 (-0.04 - +0.04)
rs73573478	153761564	0.105	0.103	9.4e-01	-0.00 (-0.02 - +0.01)	8.0e-01	+0.00 (-0.02 - +0.03)	9.2e-01	+0.00 (-0.04 - +0.04)
rs2230036	153760953	0.104	0.098	9.5e-01	+0.00 (-0.01 - +0.02)	7.0e-01	+0.00 (-0.02 - +0.03)	8.8e-01	+0.00 (-0.04 - +0.04)

Table S3. Association test results for effects independent of c.202G>A. Shown here for each SNP surveyed is: genomic position (hg19/GRCh37), DAF stratified by sex, P values and effect sizes (with 95% CI) for c.202G>A-controlled association tests under three different genetic models.
