# Supplementary material for: Genetic determinants of glucose-6-phosphate dehydrogenase activity in Kenya
Source: BMC Med Genet. 2014 Sep 9;15:93. doi: 10.1186/s12881-014-0093-6 (PMC4236593; doi:10.1186/s12881-014-0093-6)
Supplement: Additional file 6 — Linkage disequilibrium (LD) across region. Pairwise LD matrix is shown in colorimetric form, using the D’ and LOD metrics. Colors indicate: D’ = 1 and LOD ≥ 2 (red), D’ = 1 and LOD ≤ 2 (blue), D’ < 1 and LOD ≥ 2 (pink), D’ < 1 and LOD ≤ 2 (white). [file s12881-014-0093-6-S6.pdf]

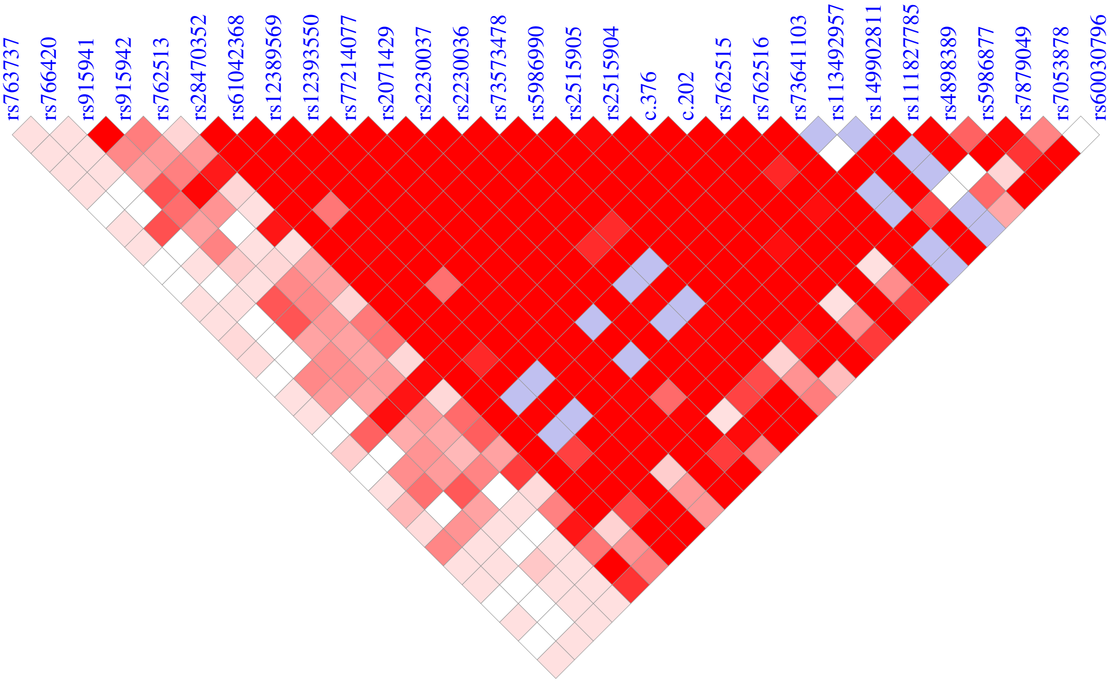

Figure S3. Linkage disequilibrium (LD) across region. Pairwise LD matrix is shown in colorimetric form, using the D' and LOD metrics. Colors indicate: D' = 1 and LOD ≥ 2 (red), D' = 1 and LOD ≤ 2 (blue), D' < 1 and LOD ≥ 2 (pink), D' < 1 and LOD ≤ 2 (white).
