# Supplementary material for: Genetic determinants of glucose-6-phosphate dehydrogenase activity in Kenya
Source: BMC Med Genet. 2014 Sep 9;15:93. doi: 10.1186/s12881-014-0093-6 (PMC4236593; doi:10.1186/s12881-014-0093-6)
Supplement: Additional file 7 — Association test results of full-length and partial haplotypes for effects independent of c.202G>A. Shown here for each haplotype surveyed is: allele identity across all sites (0=ancestral, 1=derived, _=either), population frequency, P values and effect sizes (with 95% CI) for c.202G>A-controlled association tests. [file s12881-014-0093-6-S7.pdf]

Table S4. Association test results of full-length and partial haplotypes for effects independent of c.202G>A. Shown here for each haplotype surveyed is: allele identity across all sites (0=ancestral, 1=derived, _=either), population frequency, P values and effect sizes (with 95% CI) for c.202G>A-controlled association tests.

Haplotype	Frequency	P	Effect Size
__01_0__0__1__0000_00___1_____	0.188	1.1e-06	+0.025 (+0.015 - +0.036)
100100000011000000000000111100	0.055	5.0e-05	+0.036 (+0.019 - +0.053)
101011001010001111011000011000	0.044	5.6e-04	-0.034 (-0.053 - -0.015)
011001001010001001010000011101	0.021	3.0e-03	-0.042 (-0.069 - -0.014)
111010000011000000000000111000	0.021	6.0e-03	+0.038 (+0.011 - +0.065)
__10_1__1__0__1111_11___0_____	0.219	1.6e-02	-0.016 (-0.030 - -0.003)
111000000011000000000000111100	0.016	5.5e-02	-0.030 (-0.061 - +0.001)
__10_0__0__0__0000_00___1_____	0.072	5.7e-02	-0.015 (-0.030 - +0.000)
__10_1__1__0__1001_10___0_____	0.092	9.7e-02	-0.012 (-0.025 - +0.002)
__10_0__0__0__0000_00___0_____	0.013	1.1e-01	-0.029 (-0.064 - +0.006)
100100000110000000000000111000	0.022	1.5e-01	+0.020 (-0.007 - +0.046)
110100000011000000000000111100	0.017	1.7e-01	+0.021 (-0.009 - +0.052)
111000000000000000000000100010	0.014	2.0e-01	-0.022 (-0.056 - +0.012)
110100000011000000000000111000	0.037	2.0e-01	+0.014 (-0.007 - +0.035)
000100000011000000000000111000	0.019	2.3e-01	-0.018 (-0.046 - +0.011)
__00_1__1__0__1001_10___0_____	0.015	2.3e-01	-0.020 (-0.051 - +0.012)
100100100010110000000010011100	0.014	2.4e-01	+0.020 (-0.013 - +0.054)
000101001010001111011000011000	0.024	2.4e-01	-0.015 (-0.041 - +0.010)
__01_1__1__0__1111_11___0_____	0.036	2.9e-01	-0.011 (-0.032 - +0.010)
__01_0__0__0__0000_00___0_____	0.098	3.2e-01	+0.007 (-0.007 - +0.020)
111000000000000000000000100000	0.013	3.5e-01	-0.017 (-0.051 - +0.018)
100000000110000000000000111000	0.035	3.8e-01	-0.010 (-0.031 - +0.012)
__10_0__0__1__0000_00___1_____	0.058	3.9e-01	+0.007 (-0.009 - +0.024)
010101001010001111011000011000	0.010	3.9e-01	-0.017 (-0.056 - +0.022)
000000000110000000000000111000	0.015	4.0e-01	+0.014 (-0.018 - +0.046)
010100100010110000000010011100	0.014	4.9e-01	+0.012 (-0.021 - +0.045)
001000000000000000000000110010	0.017	5.1e-01	-0.010 (-0.041 - +0.020)
100100000000000000000000100010	0.013	5.1e-01	-0.011 (-0.046 - +0.023)
000100000011000000000000111000	0.011	5.2e-01	+0.012 (-0.025 - +0.050)
101011001010001111111000011000	0.145	5.7e-01	+0.005 (-0.012 - +0.021)
000100100010110000000010011100	0.042	6.1e-01	+0.005 (-0.015 - +0.025)
__01_0__0__0__0000_00___1_____	0.083	6.1e-01	-0.004 (-0.018 - +0.011)
111001011010001001010000011000	0.017	6.9e-01	+0.006 (-0.024 - +0.037)
001011001010001111111000011000	0.017	7.6e-01	-0.005 (-0.037 - +0.027)
100100000011000000000000111000	0.019	8.0e-01	-0.004 (-0.032 - +0.025)
011001011010001001010000011000	0.043	8.4e-01	+0.002 (-0.017 - +0.021)
__01_1__1__0__1001_10___0_____	0.016	8.6e-01	+0.003 (-0.028 - +0.034)
__00_0__0__0__0000_00___1_____	0.063	9.7e-01	+0.000 (-0.016 - +0.017)
