# Supplementary material for: Genetic determinants of glucose-6-phosphate dehydrogenase activity in Kenya
Source: BMC Med Genet. 2014 Sep 9;15:93. doi: 10.1186/s12881-014-0093-6 (PMC4236593; doi:10.1186/s12881-014-0093-6)
Supplement: Additional file 8 — Allele sharing with H+ or H- explains variance independent of c.202G>A. G6PD activity as stratified by sex and haplogroup, where the latter is defined by categorizing each individual according to extensive allele sharing with either the H+, H-, or neither haplotype (see Methods). In box-and-whisker plots, center of box represents median, edges of box represent upper and lower quartile bounds, and whiskers represent either minimum and maximum values or 1.5 times the interquartile range, whichever is less extreme. The P-value displayed in the figure represents the statistical significance of allele sharing under a linear model which incorporates c.202G>A genotype and gender as covariates (NB: sex-stratified testing reveals Pmale=8.8×10^−5^ and Pfemale=6.2×10^−4^). [file s12881-014-0093-6-S8.pdf]

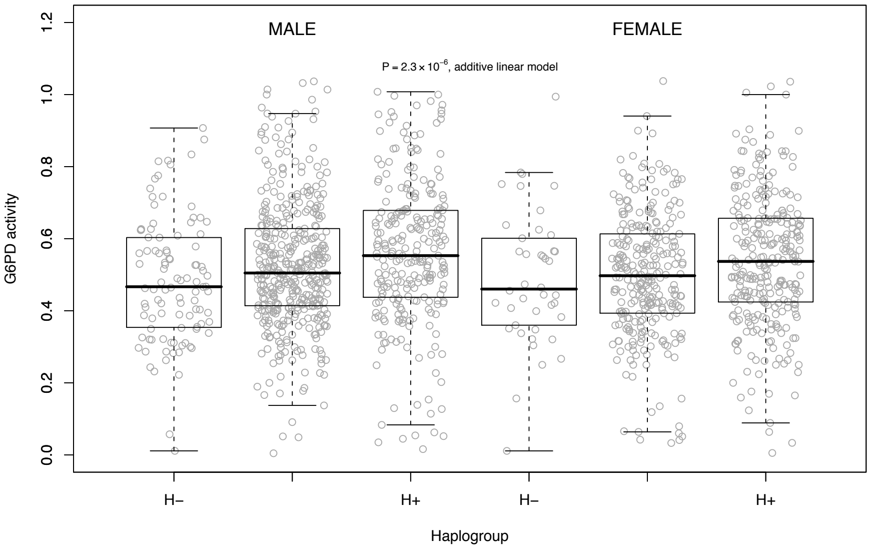

Figure S4. Allele sharing with H+ or H- explains variance independent of c.202G>A. G6PD activity as stratified by sex and haplogroup, where the latter is defined by categorizing each individual according to extensive allele sharing with either the H+, H-, or neither haplotype (see Methods). In box-and-whisker plots, center of box represents median, edges of box represent upper and lower quartile bounds, and whiskers represent either minimum and maximum values or 1.5 times the interquartile range, whichever is less extreme. The P-value displayed in the figure represents the statistical significance of allele sharing under a linear model which incorporates c.202G>A genotype and gender as covariates (NB: sex-stratified testing reveals $P_{male} = 8.8 \times 10^{-5}$ and $P_{female} = 6.2 \times 10^{-4}$).
