# Supplementary material for: Genetic determinants of glucose-6-phosphate dehydrogenase activity in Kenya
Source: BMC Med Genet. 2014 Sep 9;15:93. doi: 10.1186/s12881-014-0093-6 (PMC4236593; doi:10.1186/s12881-014-0093-6)
Supplement: Additional file 9 — Defining qualitative G6PD deficiency. The intermodal minimum in the G6PD activity distribution in males was determined using kernel density estimation of the probability density function (red) and provided our ad hoc cutoff (blue) for distinguishing normal vs. deficient. A histogram of the G6PD activity distribution in males is superimposed for clarity. [file s12881-014-0093-6-S9.pdf]

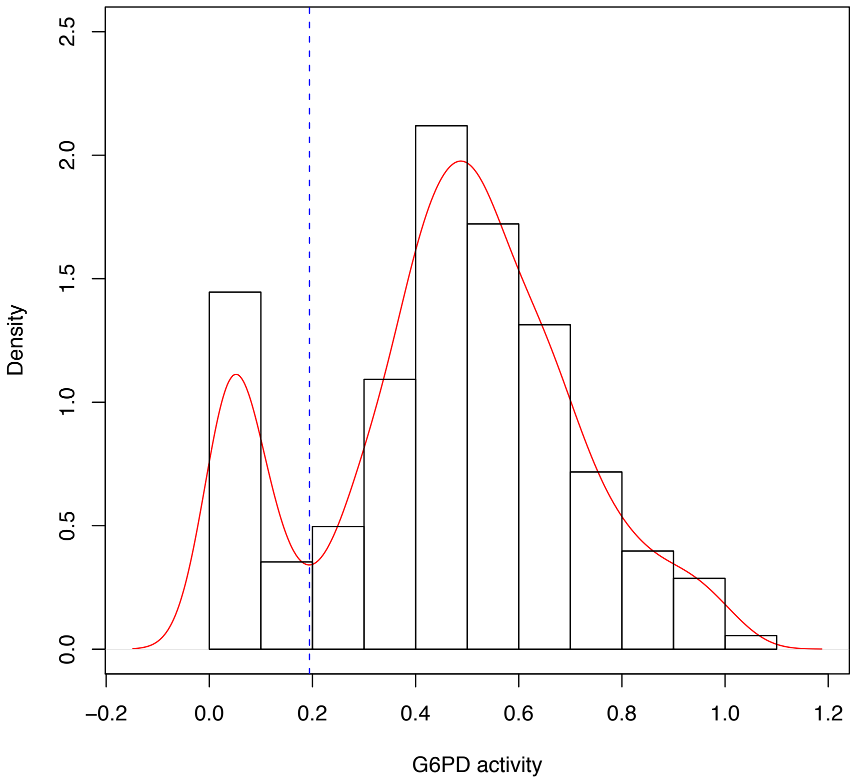

Figure S5. Defining qualitative G6PD deficiency. The intermodal minimum in the G6PD activity distribution in males was determined using kernel density estimation of the probability density function (red) and provided our ad hoc cutoff (blue) for distinguishing normal vs. deficient. A histogram of the G6PD activity distribution in males is superimposed for clarity.
